# Depletion of dendritic cells enhances susceptibility to cell-free but not cell-associated HTLV-1 infection in CD11c-DTR transgenic mice

**DOI:** 10.1186/1742-4690-8-S1-A25

**Published:** 2011-06-06

**Authors:** Saifur Rahman, Sharrón L Manuel, Zafar K Khan, Brian Wigdahl, Frederic Tangy, Pooja Jain

**Affiliations:** 1 Department of Microbiology and Immunology, and the Drexel Institute for Biotechnology and Virology Research, Drexel University College of Medicine, Doylestown, PA, 18902, USA; 2 Department of Microbiology and Immunology, and the Institute for Molecular Medicine and Infectious Disease, Drexel University College of Medicine, Philadelphia, PA, 19102, USA; 3 Unité de Génomique Virale et Vaccination, URA-3015, Centre National de la Recherche Scientifique (CNRS), Institut Pasteur, Paris, France

## 

Human T-cell leukemia virus type 1 (HTLV-1) is associated with two immunologically distinct diseases: HTLV-1-associated myelopathy/tropical spastic paraparesis (HAM/TSP) and adult T-cell leukemia (ATL). The genesis of these diseases is believed to be associated with the route (mucosa versus blood), mode (cell-free versus cell-associated) of primary infection and eventual modulation of dendritic cell (DC) functions. To explore the role of DCs during early HTLV-1 infection in vivo, we used a chimeric HTLV-1 with a replaced envelope gene from Moloney murine leukemia virus to allow HTLV-1 to fuse with murine cells, which are generally not susceptible to infection with human retroviruses. We also used a CD11c-DTR transgenic mouse model system that permits the conditional transient depletion of murine CD11c+ DCs. We infected these transgenic mice with HTLV-1 using both cell-free and cell-associated infection routes in the absence and presence of DCs. The ablation of DCs led to an enhanced susceptibility to infection with cell-free, but not cell-associated HTLV-1 in both the CD4 and non-CD4 fractions, as measured by the proviral load. Infection with cell-free virus in the absence of DCs was also found to have increased levels of Tax mRNA. Moreover, the depletion of DCs was found to significantly dampen the cellular immune response (IFN-γ+CD8+ T cells) against both cell-free and cell-associated virus. These results uniquely differentiate the involvement of DCs in early cell-free compared with late cell-associated HTLV-1 infection and highlight a significant aspect of viral immunopathogenesis related to the progression of ATL and HAM/TSP after the initial infection.

